# A rare de novo nonsense mutation in *OTX2* causes early onset retinal dystrophy and pituitary dysfunction

**Published:** 2009-11-21

**Authors:** Robert H. Henderson, Kathleen A. Williamson, Joanna S. Kennedy, Andrew R. Webster, Graham E. Holder, Anthony G. Robson, David R. FitzPatrick, Veronica van Heyningen, Anthony T. Moore

**Affiliations:** 1 Moorfields Eye Hospital, London, United Kingdom; 2 UCL Institute of Ophthalmology, London, United Kingdom; 3 MRC Human Genetics Unit, Western General Hospital, Edinburgh, Scotland

## Abstract

**Purpose:**

To describe the clinical findings of a patient with an early onset retinal dystrophy and a novel mutation in *OTX2,* and to compare these findings with previously reported cases.

**Methods:**

Using direct sequencing, we screened 142 patients, who had either Leber congenital amaurosis (LCA) or early onset retinal dystrophy (EORD), for mutations in *OTX2.* All patients received a detailed ophthalmic examination including electroretinography and retinal imaging.

**Results:**

Only one mutation in *OTX2* was identified. A novel heterozygous p.S138X stop mutation was identified in a seven-year-old male who had an infantile onset retinal dystrophy. The mutation was not present in either parent or in 181 blood donor samples. There was a history of failure to thrive in infancy, poor feeding, and growth hormone deficiency. Poor vision and nyctalopia was present from the first year. Funduscopy revealed a hyperpigmented peripapillary ring with a fine granular pigmentation of the RPE throughout the fundus. The scotopic bright flash ERG a-wave was subnormal and the waveform electronegative, in keeping with dysfunction both at the level of the photoreceptor and post-phototransduction. Visual function has been stable to date.

**Conclusions:**

Mutations in *OTX2* have been reported in association with major developmental malformations of the eye, with retinal dystrophies such as LCA, and with pituitary dysfunction and seizure activity in some cases. This case adds further support for a role of *OTX2* both in retinal development and pituitary function, and highlights a novel retinal dystrophy phenotype seen in association with mutations in *OTX2.*

## Introduction

The orthodenticle protein homolog 2 (*OTX2*) is a homeobox gene that plays a critical role in retinal photoreceptor development. The gene, on chromosome 14q23.1, is organized into five exons, only three of which are translated. It contains a highly conserved homeodomain. *OTX2* is expressed during development in the neuroepithelium of most of the forebrain and midbrain, including the eye domain [1]. Complete elimination of *OTX2* function in mice by gene targeting results in the absence of the forebrain and embryonic lethality [2,3]. While *Otx2* null embryos display a severe cranial phenotype, lacking the anterior neuroectoderm, and with abnormalities in body plan [2,3], heterozygous deletion of *Otx2* leads to a variable phenotype that is dependent on genetic background. *Otx2*^+/−^ mice can be normal, have developmental eye anomalies including anterior segment malformations, severe eye abnormalities such as microphthalmia or anophthalmia, or head abnormalities [2,4]. In early development *Otx2*, specifies the anterior neuroectoderm during gastrulation [2] and is expressed throughout the forebrain and midbrain in the developing embryo [1]. Once eye development is specified, expression of *Otx2* narrows. It is required for the development of the presumptive retinal pigment epithelium (RPE) [5], where it has a continuing maintenance role throughout adulthood [6]. Analysis of *Otx2*^−/−^ mice has shown that it is essential for specification of the RPE; loss of OTX2 function results in the outer layer of the optic cup developing into ectopic neural retina [7]. However, OTX2 is also required for the development and maintenance of the neural retina. Otx2 protein is found in bipolar and in ganglion cells [6,8,9], while in the outer nuclear layer it regulates the expression of the closely related *Crx* gene that controls the expression of a suite of photoreceptor function genes, including opsins. Otx2 and Crx can also bind to common DNA-binding sequences [10]. Ablation of *OTX2* in retinal progenitor cells has been shown to affect RPE development and leads to a significant decrease in rod photoreceptors and bipolar cells, suggesting that OTX2 is also involved in retinal bipolar cell development [8]. It has recently been demonstrated that OTX2 protein is transported from the retina to the visual cortex in the developing rat, and appears to modify postnatal synaptic plasticity [9].

*OTX2* mutations in humans have been associated with a range of ophthalmological phenotypes including anophthalmia, microphthalmia, developmental anomalies of the optic nerve and chiasm, and Leber congenital amaurosis (LCA). As OTX2 plays a key role in retinal development, and a mutation in *OTX2* has been reported in association with an infantile onset retinal dystrophy [11], we screened a large panel of patients with LCA and other forms of childhood onset retinal dystrophy for mutations in this gene.

## Methods

A panel of DNA from 142 patients with LCA and severe childhood onset retinal dystrophy were recruited at Moorfields Eye Hospital and The Hospital for Children, Great Ormond Street, London, as part of an ongoing molecular genetic study of childhood retinal dystrophies. The research has local research ethics committee approval and conformed to the tenets of the Declaration of Helsinki. A full clinical history was taken and examination performed in all subjects. In older children and adults this included, where possible, best corrected logMAR visual acuity and color vision (Hardy Rand Rittler, Richmond Products, Albuquerque, NM); slit-lamp biomicroscopy or indirect ophthalmoscopy; Goldmann visual fields; autorefraction (Luneau L62–3D autorefractokeratometer - LUNEAU Chartres, France), axial length measurement, and auto-keratometry (IOL Master, Carl Zeiss Meditec, Dublin, CA); optical coherence tomography (Stratus OCT™ - software version 3; Carl Zeiss Meditec, Inc.); fundus photography (TRC-50IX with IMAGEnet 2000 system software–TOPCON Corporation, Japan), and fundus autofluorescence (Heidelberg Retinal Angiograph-II, Heidelberg Engineering, Heidelberg, Germany). All patients had electroretinography performed either at our institutions or at the referring hospital. Most patients had tests performed at diagnosis which, in the older patients, predated the ISCEV standards. Patients seen more recently, or in whom we wished to confirm the diagnosis, had electroretinography conducted in accordance with current International Society for Clinical Electrophysiology of Vision standards using gold foil or DTL electrodes [12,13]. ERG testing in infants and young children was performed using skin electrodes in accordance with previously published protocols [14,15].

DNA was extracted from whole blood obtained from each affected individual and immediate family members using the Nucleon BACC-2 kit (GE Healthcare) performed as per manufacturer’s instructions. DNA samples from all patients were sent to Asper Ophthalmics (Tartu, Estonia) for analysis using the LCA chip [16]. This is a microarray which contained (at the time of the study) 344 disease associated SNPs and several common variant polymorphisms in six LCA associated genes and 2 early onset genes (*GUCY2D, CRX, RPE65, CRB1, RPGRIP1, AIPL1, LRAT,* and *MERTK*). Genorama genotyping software (Asper Ophthalmics Ltd) can subsequently produce a bidirectional allele call at each SNP being interrogated. The panel subsequently underwent direct sequencing of PCR products [11] performed using an ABI 3130 36 cm capillary sequencer (Applied Biosystems, Inc., Foster City, CA). 181 control DNA samples isolated from anonymous Aberdeen-based blood donors were screened for DNA mismatches using the Idaho Technology Inc. Light Scanner (Salt Lake City, UT). Fragments revealing a nucleotide mismatch were fully sequenced to identify the mutations.

## Results

**Figure 1:**
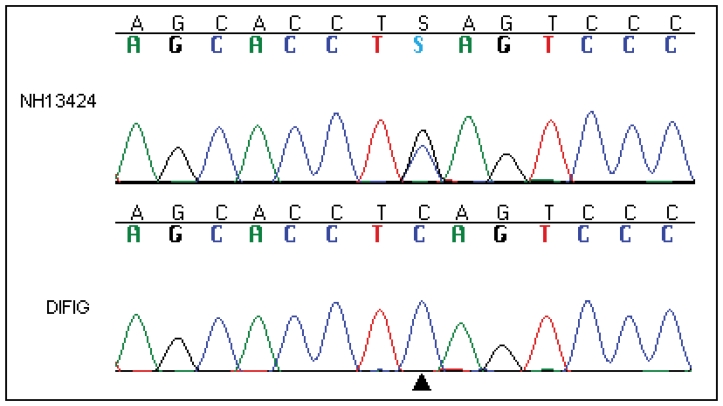
Direct sequencing. At the position indicated by the black arrowhead, the heterozygous C>G change is revealed in the DNA sequence trace for patient NH13424. Below this the wild type trace for normal control subject DIFIG is shown.

A de novo heterozygous c.413C>G p.S138X stop mutation was identified in a seven-year-old male (Figure 1; Pt 13424) who was negative for all variants assayed by the LCA chip. The mutation was not present in either parent. This mutation was not detected in 181 controls. Known polymorphic single nucleotide polymorphisms (SNPs), were observed at similar frequencies in the disease cohort and the blood donor controls, indicating that the ethnic background of the patient cohort and the controls was comparable.

The patient had a history of failure to thrive from birth and multiple investigations of the possible cause including investigations for Batten disease, Williams syndrome, and Russell-Silver dwarfism, all of which were negative. At 9 months old, required nasogastric feeding for 6 months following a history of failure to thrive. He has always been on the lowest percentiles for stature. Growth hormone deficiency was suggested indirectly via low levels of IGF1 and IGFBP3. An MRI scan was reported as normal. His parents noted poor vision and nyctalopia during his first year of life. There was no family history of eye disease. One younger sibling who has no visual problems was unavailable for further investigation.

**Figure 2:**
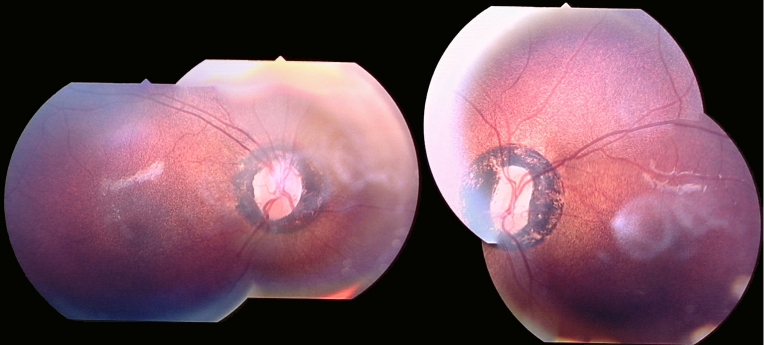
Fundus photography. Images are right and left eye color fundus photograph composites of patient NH13424 performed using TOPCON retinal camera at 2.45x magnification at 35 degrees. Composite images were created using Adobe Photoshop. Image cropping and reflections are observed secondary to poor pupil dilatation. The images show significant and abnormal peripapillary hyperpigmentation with fine granular pigmentation at the level of the retinal pigment epithelium (RPE).

Ophthalmic examination when he was six years old disclosed a small angle alternating esotropia with mild torsional nystagmus. Visual acuities were 0.4 and 0.5 logMAR in his right and left eyes, respectively. Cycloplegic retinoscopy revealed a refractive error of 0.00 diopter sphere/+3.00 diopter cylinder at 180 degrees in the right eye, and +3.00 diopter sphere /+2.50 diopter cylinder at 180 degrees in the left. He had normal color vision, according to Hardy Rand Rittler; he had normal pupil reactions and clear lenses. Funduscopy revealed a hyperpigmented area of retina surrounding the optic disc with a fine granular pigmentation of the RPE throughout the fundus (Figure 2). There was no vascular attenuation.

**Figure 3:**
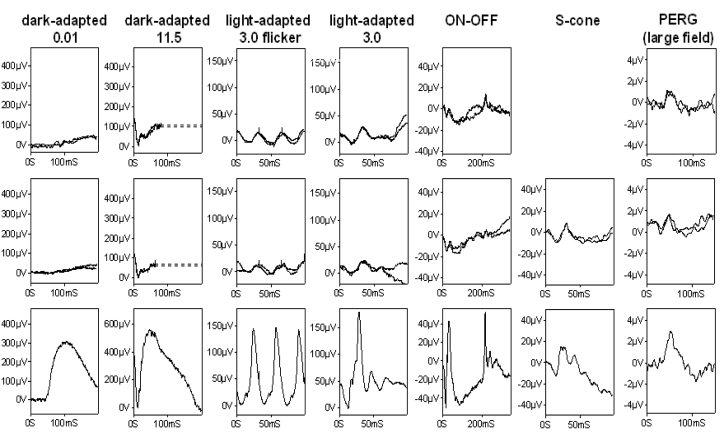
Electrophysiology. Full-field ERGs and pattern ERGs from the right (row 1) and left eye (row 2) of the patient and normal examples (row 3) for comparison. Full-field ERGs were performed using corneal electrodes in August 2007; pattern ERGs had been recorded to a 30 degree field checkerboard stimulus using periorbital surface electrodes two years earlier. Dark-adapted ERGs are shown for flash intensities of 0.01 and 11.5 cd.s.m^−2^; light-adapted ERGs for a flash intensity of 3.0 cd.s.m^−2^ at 30 Hz (flicker) and at 2 Hz.. ON-OFF ERGs used an orange stimulus (560 cd.m^−2^, duration 200 ms) superimposed on a green background (150 cd.m^−2^). S-cone ERGs used a blue stimulus (445 nm, 80 cd.m^−2^) on an orange background (620 nm, 560 cd.m^−2^). Eye movement artifacts are replaced by broken lines for clarity. S-cone ERGs were unavailable for the right eye.

ERGs were performed according to international standards [13]. The dark-adapted bright flash ERG was markedly electronegative with additional a-wave reduction. Light-adapted flicker ERGs were delayed and subnormal and the single flash cone ERG was subnormal with a reduced b:a ratio (Figure 3). The profoundly negative ERG suggests dysfunction that is both marked and at a level that is post-phototransduction, usually post-receptoral. The a-wave was subnormal but not delayed, indicating additional loss of photoreceptor function. The ON-OFF and S-cone ERGs [17,18] suggesting greater ON- than OFF- bipolar pathway involvement. The pattern ERG P50 component was reduced bilaterally, which is consistent with macular involvement. (Figure 3)

## Discussion

This study provides evidence of a novel mutation in OTX2 associated with early onset retinal dystrophy and pituitary insufficiency. This mutation is predicted to lead a truncated protein. Chatelain et al. [19] observed that OTX2 truncation at residues 106 and 161, flanking the site of the predicted termination, described here, leads to loss of transactivation function. This strongly suggests that the mutation identified in our patient is also likely to cause haploinsufficiency.

Clinical studies have demonstrated that mutations in *OTX2* are associated with a wide range of ocular phenotypes (Table 1) [7,11,20–22]. Experimentally, the role of *OTX2* in regional specification of the eye, particularly the RPE, has long been established from expression studies in normal and mutant animal models [1,4,23–26]. Subsequent studies have also demonstrated the role of *OTX2* in the development of both the retinal photoreceptors and bipolar cells [25,27]. It has been hypothesized that *OTX2* is involved in retinal photoreceptor cell fate but not in bipolar cell fate in early development, while it is involved in the terminal differentiation of both photoreceptors and bipolar cells late in development in cooperation with *CRX* [8]. Pituitary dysfunction has been described in two patients with heterozygous 14q22–23 microdeletions involving *OTX2* [28,29]. Recently, the role of OTX2 in pituitary development has been further delineated with three case reports of *OTX2* mutations associated with pituitary hormone deficiency (CPHD) [21,22]. A further case of anophthalmia associated with an *OTX2* mutation was reported to have isolated growth hormone deficiency and short stature [7]. In the latter publication a requirement for OTX2 binding was postulated at a site previously shown to lie in the promoter of HESX1 [30].

**Table 1:** Previously published mutations in *OTX2* with associated phenotypes.

**Nucleotide**	**Protein**	**Phenotype**	**Reference**
c.674A>G	p.Asn225Ser	CPHD	[22]
c.674A>G	p.Asn225Ser	CPHD	[22]
whole gene deletion	whole gene deletion	Extreme microphthalmia	[20]
whole gene deletion	whole gene deletion	Anophthalmia	[20]
c.93C>G	p.Tyr31X	Microphthalmia	[20]
c.106dupC	p.Arg36ProfsX52	Microphthalmia OD/ normal OS	[20]
c.106dupC	p.Arg36ProfsX52	Anophthalmia OD/ Coloboma OS	[20]
c.289C>T	p.Gln97X	Extreme microphthalmia	[20]
c.289C>T	p.Gln97X	Inferior iris coloboma OD/ retinal coloboma OS	[20]
c.373_374delAG	p.Gly126TrpfsX11	Anophthalmia	[20]
c.404_405dupCT	p.Ser136LeufsX43	Anophthalmia and CPHD	[21]
c.402dupC	p.Ser135LeufsX2	Anophthalmia, GH deficiency; cleft palate	[7]
c.463_464dupGC	p.Ser156LeufsX23	bilateral anophthalmia; developmental delay	[11]
c.265C>G	p.Arg89Gly	bilateral microphthalmia	[11]
c.81delC	p.Ser28ProfsX23	bilateral severe microphthalmia	[11]
c.537T>A	p.Tyr179X	severe microphthalmia and colobomata, developmental delay seizures	[11]
c.537T>A	p.Tyr179X	bilateral mild microphthalmia, retinal dystrophy originally described as LCA	[11]
c.117_118delCC	p.Arg40GlyfsX47	bilateral anophthalmia	[11]
c.295C>T	p.Gln99X	bilateral anopthalmia	[11]
c.397C>A	p.Pro133Thr	bilateral microphthalmia	[11]
c.400C>G	p.Pro134Ala	anophthalmia OS/ OD normal	[11]

Mutations in *OTX2*. a summary of the published cases. Table lists previously published mutations in *OTX2* with associated phenotypes. The nucleotide and protein nomenclature used conforms to current standards and may differ from the notation used in the original publications (LOVD). Abbreviations: CPHD represents combined pituitary hormone deficiency; OD represents right eye; OS represents left eye; GH represents growth hormone.

The phenotypic spectrum described in this case report is consistent with the assigned multiple roles of *OTX2* in the development and function of both RPE and neural retinal, as well as in the pituitary. Mutations in *OTX2* are a rare cause of infantile onset retinal dystrophies and the finding of an electronegative ERG with additional a-wave reduction suggests that there is dysfunction at the level of the photoreceptor and also post-phototransduction, likely to involve both ON- and OFF- bipolar cells. Funduscopy revealed a fine granular pigmentation of the RPE. In addition, an unusual hyperpigmented juxtapapillary ring, not normally seen in retinal dystrophies, was observed. The history of early failure to thrive and subsequent short stature with low implied growth hormone levels adds further support for a role for *OTX2* in pituitary function.

## References

[1] Simeone A Acampora D Mallamaci A Stornaiuolo A D'Apice MR Nigro V Boncinelli E A vertebrate gene related to orthodenticle contains a homeodomain of the bicoid class and demarcates anterior neuroectoderm in the gastrulating mouse embryo. EMBO J 1993 12 2735 47 8101484 10.1002/j.1460-2075.1993.tb05935.x PMC413524

[2] Acampora D Mazan S Lallemand Y Avantaggiato V Maury M Simeone A Brûlet P Forebrain and midbrain regions are deleted in Otx2−/− mutants due to a defective anterior neuroectoderm specification during gastrulation. Development 1995 121 3279 90 7588062 10.1242/dev.121.10.3279

[3] Ang SL Jin O Rhinn M Daigle N Stevenson L Rossant J A targeted mouse Otx2 mutation leads to severe defects in gastrulation and formation of axial mesoderm and to deletion of rostral brain. Development 1996 122 243 52 8565836 10.1242/dev.122.1.243

[4] Matsuo I Kuratani S Kimura C Takeda N Aizawa S Mouse Otx2 functions in the formation and patterning of rostral head. Genes Dev 1995 9 2646 58 7590242 10.1101/gad.9.21.2646

[5] Martínez-Morales JR Dolez V Rodrigo I Zaccarini R Leconte L Bovolenta P Saule S OTX2 activates the molecular network underlying retina pigment epithelium differentiation. J Biol Chem 2003 278 21721 31 12663655 10.1074/jbc.M301708200

[6] Rath MF Morin F Shi Q Klein DC Moller M Ontogenetic expression of the Otx2 and Crx homeobox genes in the retina of the rat. Exp Eye Res 2007 85 65 73 17467693 10.1016/j.exer.2007.02.016

[7] Dateki S Fukami M Sato N Muroya K Adachi M Ogata T OTX2 mutation in a patient with anophthalmia, short stature, and partial growth hormone deficiency: functional studies using the IRBP, HESX1, and POU1F1 promoters. J Clin Endocrinol Metab 2008 93 3697 702 18628516 10.1210/jc.2008-0720

[8] Koike C Nishida A Ueno S Saito H Sanuki R Sato S Furukawa A Aizawa S Matsuo I Suzuki N Kondo M Furukawa T Functional roles of Otx2 transcription factor in postnatal mouse retinal development. Mol Cell Biol 2007 27 8318 29 17908793 10.1128/MCB.01209-07 PMC2169187

[9] Sugiyama S Prochiantz A Hensch TK From brain formation to plasticity: insights on Otx2 homeoprotein. Dev Growth Differ 2009 51 369 77 19298552 10.1111/j.1440-169X.2009.01093.x

[10] Esumi N Kachi S Hackler L Jr Masuda T Yang Z Campochiaro PA Zack DJ BEST1 expression in the retinal pigment epithelium is modulated by OTX family members. Hum Mol Genet 2009 18 128 41 18849347 10.1093/hmg/ddn323 PMC2605189

[11] Ragge NK Brown AG Poloschek CM Lorenz B Henderson RA Clarke MP Russell-Eggitt I Fielder A Gerrelli D Martinez-Barbera JP Ruddle P Hurst J Collin JR Salt A Cooper ST Thompson PJ Sisodiya SM Williamson KA Fitzpatrick DR van Heyningen V Hanson IM Heterozygous mutations of OTX2 cause severe ocular malformations. Am J Hum Genet 2005 76 1008 22 15846561 10.1086/430721 PMC1196439

[12] Holder GE Brigell MG Hawlina M Meigen T Vaegan, Bach M. ISCEV standard for clinical pattern electroretinography–2007 update. Doc Ophthalmol 2007 114 111 6 17435967 10.1007/s10633-007-9053-1 PMC1896293

[13] Marmor MF Fulton AB Holder GE Miyake Y Brigell M Bach M ISCEV Standard for full-field clinical electroretinography (2008 update). Doc Ophthalmol 2009 118 69 77 19030905 10.1007/s10633-008-9155-4

[14] Holder GE, Robson AG. Paediatric Electrophysiology: A Practical Approach. In: Lorenz B, Moore AT, editors. Paediatric Ophthalmology, Neuro-ophthalmology, Genetics. Heidelberg: Springer; 2006: 133–55.

[15] Kriss A, Thompson D. Visual Electrophysiology. In: Taylor D, editor. Paediatric ophthalmology. Oxford: Blackwell; 1997. p. 93–121.

[16] Henderson RH Waseem N Searle R van der Spuy J Russell-Eggitt I Bhattacharya SS Thompson DA Holder GE Cheetham ME Webster AR Moore AT An assessment of the apex microarray technology in genotyping patients with leber congenital amaurosis and early-onset severe retinal dystrophy. Invest Ophthalmol Vis Sci 2007 48 5684 9 18055820 10.1167/iovs.07-0207

[17] Sieving PA Photopic ON- and OFF-pathway abnormalities in retinal dystrophies. Trans Am Ophthalmol Soc 1993 91 701 73 8140708 PMC1298484

[18] Audo I Michaelides M Robson AG Hawlina M Vaclavik V Sandbach JM Neveu MM Hogg CR Hunt DM Moore AT Bird AC Webster AR Holder GE Phenotypic variation in enhanced S-cone syndrome. Invest Ophthalmol Vis Sci 2008 49 2082 93 18436841 10.1167/iovs.05-1629

[19] Chatelain G Fossat N Brun G Lamonerie T Molecular dissection reveals decreased activity and not dominant negative effect in human OTX2 mutants. J Mol Med 2006 84 604 15 16607563 10.1007/s00109-006-0048-2

[20] Wyatt A Bakrania P Bunyan DJ Osborne RJ Crolla JA Salt A Ayuso C Newbury-Ecob R Abou-Rayyah Y Collin JR Robinson D Ragge N Novel heterozygous OTX2 mutations and whole gene deletions in anophthalmia, microphthalmia and coloboma. Hum Mutat 2008 29 E278 83 18781617 10.1002/humu.20869

[21] Tajima T Ohtake A Hoshino M Amemiya S Sasaki N Ishizu K Fujieda K OTX2 loss of function mutation causes anophthalmia and combined pituitary hormone deficiency with a small anterior and ectopic posterior pituitary. J Clin Endocrinol Metab 2009 94 314 9 18854396 10.1210/jc.2008-1219

[22] Diaczok D Romero C Zunich J Marshall I Radovick S A novel dominant negative mutation of OTX2 associated with combined pituitary hormone deficiency. J Clin Endocrinol Metab 2008 93 4351 9 18728160 10.1210/jc.2008-1189 PMC2582563

[23] Zuber ME Gestri G Viczian AS Barsacchi G Harris WA Specification of the vertebrate eye by a network of eye field transcription factors. Development 2003 130 5155 67 12944429 10.1242/dev.00723

[24] Martinez-Morales JR Signore M Acampora D Simeone A Bovolenta P Otx genes are required for tissue specification in the developing eye. Development 2001 128 2019 30 11493524 10.1242/dev.128.11.2019

[25] Larsen KB Lutterodt M Rath MF Møller M Expression of the homeobox genes PAX6, OTX2, and OTX1 in the early human fetal retina. Int J Dev Neurosci 2009 27 485 92 19414065 10.1016/j.ijdevneu.2009.04.004

[26] Hever AM Williamson KA van Heyningen V Developmental malformations of the eye: the role of PAX6, SOX2 and OTX2. Clin Genet 2006 69 459 70 16712695 10.1111/j.1399-0004.2006.00619.x

[27] Kim DS Matsuda T Cepko CL A core paired-type and POU homeodomain-containing transcription factor program drives retinal bipolar cell gene expression. J Neurosci 2008 28 7748 64 18667607 10.1523/JNEUROSCI.0397-08.2008 PMC2714707

[28] Elliott J Maltby EL Reynolds B A case of deletion 14(q22.1→q22.3. associated with anophthalmia and pituitary abnormalities. J Med Genet 1993 30 251 2 7682620 10.1136/jmg.30.3.251 PMC1016311

[29] Nolen LD Amor D Haywood A St Heaps L Willcock C Mihelec M Tam P Billson F Grigg J Peters G Jamieson RV Deletion at 14q22–23 indicates a contiguous gene syndrome comprising anophthalmia, pituitary hypoplasia, and ear anomalies. Am J Med Genet A 2006 140 1711 8 16835935 10.1002/ajmg.a.31335

[30] Spieler D Bäumer N Stebler J Köprunner M Reichman-Fried M Teichmann U Raz E Kessel M Wittler L Involvement of Pax6 and Otx2 in the forebrain-specific regulation of the vertebrate homeobox gene ANF/Hesx1. Dev Biol 2004 269 567 79 15110720 10.1016/j.ydbio.2004.01.044

